# Ultrastable liquid crystalline blue phase from molecular synergistic self-assembly

**DOI:** 10.1038/s41467-021-21564-y

**Published:** 2021-03-04

**Authors:** Wei Hu, Ling Wang, Meng Wang, Tingjun Zhong, Qian Wang, Lanying Zhang, Feiwu Chen, Kexuan Li, Zongcheng Miao, Dengke Yang, Huai Yang

**Affiliations:** 1 Beijing Advanced Innovation Center for Materials Genome Engineering & Department of Materials Science and Engineering College of Engineering, Peking University, Beijing, PR China; 2 Department of Chemistry and Chemical Engineering, School of Chemistry and Biological Engineering, University of Science and Technology Beijing, Beijing, PR China; 3 School of Materials Science and Engineering, Tianjin University, Tianjin, PR China; 4 School of Science, Xijing University, Xi’an, Shannxi PR China; 5 Chemical Physics Interdisciplinary Program in Liquid Crystal Institute, Kent State University, Kent, OH USA

**Keywords:** Liquid crystals, Photonic crystals, Molecular self-assembly, Organic molecules in materials science

## Abstract

Fabricating functional materials via molecular self-assembly is a promising approach, and precisely controlling the molecular building blocks of nanostructures in the self-assembly process is an essential and challenging task. Blue phase liquid crystals are fascinating self-assembled three-dimensional nanomaterials because of their potential information displays and tuneable photonic applications. However, one of the main obstacles to their applications is their narrow temperature range of a few degrees centigrade, although many prior studies have broadened it to tens via molecular design. In this work, a series of tailored uniaxial rodlike mesogens disfavouring the formation of blue phases are introduced into a blue phase system comprising biaxial dimeric mesogens, a blue phase is observed continuously over a temperature range of 280 °C, and the range remains over 132.0 °C after excluding the frozen glassy state. The findings show that the molecular synergistic self-assembly behavior of biaxial and uniaxial mesogens may play a crucial role in achieving the ultrastable three-dimensional nanostructure of blue phases.

## Abstract (web summary)

Blue phases are spatially ordered yet fragile liquid crystalline structures, bearing applications in optoelectronics and photonics. Hu et al. show that self-assembly within a mixture of different mesogens may significantly broaden the temperature range over which they are stable.

## Introduction

Blue phase liquid crystals (BPLCs) have self-assembled three-dimensional cubic defect structures originating from the competition between the packing topology and chiral forces^1^. In BPs, mesogenic molecules are known to exhibit a “double-twist” arrangement along the *x*- and *y*-axes, and such a unique structure is called a double-twisted cylinder (DTC)^2–4^. In the space between the DTCs, the molecular orientation cannot pack continuously, resulting in the formation of energetically disfavoured disclinations within the cubic lattices^3,5^. As a result, BPs usually exist in a narrow temperature range of 0.5–2.0 °C between an isotropic (Iso) and a cholesteric (Ch) phase in highly chiral liquid crystals. Three types of BPs, BP III, BP II, and BP I, are observed in sequence upon cooling. BP I and BP II possess body-centered cubic (BCC) and simple cubic (SC) structures, respectively, whereas BP III is considered amorphous in nature^3^. To obtain a BP with a wide temperature range, various strategies have been proposed, such as introducing polymers^1,6^ and nanoparticles^7,8^ as well as molecular design^9–18^. For example, Kikuchi et al. pioneered a polymer stabilized BP (PSBP) system in which the BPs were stabilized by polymer networks, obtaining a wide temperature range of over 60.0 °C (~53.0 to −13.0 °C)^1^. This PSBP was applied by the Samsung Co. in 2008 to demonstrate the first BP liquid crystal display prototype^19^, with attractive characteristics^20^ such as a submillisecond grey-to-grey response time, color filter- and alignment layers-free operation, optically isotropic voltage-off state, and large cell gap tolerance. Subsequently, Castles et al. achieved a BP composite material with a superwide temperature range (from −125 to 125 °C observed via polarizing optical microscopy) by injecting an achiral LC mixture with a high clearing point into a polymer template prefabricated from a PSBP in which LC was washed-out^6^. This type of BP templated material has considerable potential in photonics applications, such as mirrorless lasers and switchable electro-optic devices^21^. On the other hand, developing low molecular weight BPLCs, such as those comprising well-designed molecules with special configurations (dimeric molecules^10^, U-shaped molecules^12,15,16^, T-shaped molecules,^11^ or bent-core molecules^9,13,14^), could be another promising pathway to obtaining BPs with a wide temperature range. The landmark work of Coles et al. broadened the temperature range to 44.0 °C, from 60.0 to 16.0 °C, using liquid crystalline dimers^10^, in which molecular biaxiality and flexoelectricity played a crucial role in the formation of stable DTC structures^5^. Furthermore, Yang et al. reported a hydrogen-bonding stabilized BP over a wide temperature range and found that weak interactions from fluorinated molecules were beneficial for the formation of stable cubic nanostructures^17^. However, to our knowledge, BPLCs covering the temperature range from 80.0 to −30.0 °C, which is considered the working temperature range of practical LC devices, have not been reported in low molecular weight LC systems to date. In this work, we demonstrate that an ultrastable BP system can be achieved by engineering the molecular synergistic self-assembly of tailored biaxial dimeric and uniaxial rodlike mesogens.

## Results

### Molecular design and materials combination

**Fig. 1:**
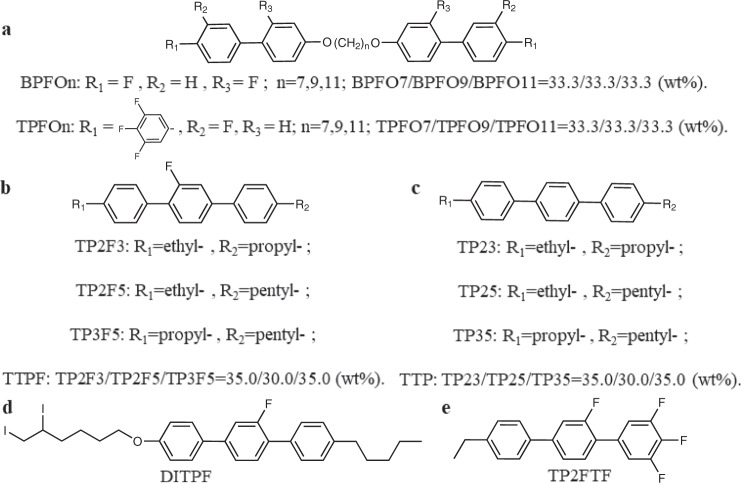
The molecular structures of the materials. **a** Mixtures of BPFOn and TPFOn were made from dimeric LCs with fluoro substituents. **b** Mixture TTPF from rodlike LCs with a single lateral fluoro substituent. **c** Mixture analogs of TTPF without a lateral fluoro substituent named TTP used for contrast with TTPF. **d** An analog of TTPF with double iodine substituents named DITPF used for SAXS characterization. **e** An analog of TTPF with three terminal groups of fluoro substituents named TP2FTF used to improve the polarity of the materials for better electro-optical performance.

The chemical structures of the mesogens used in this work are shown in Fig. 1. A series of biaxial dimers with two rigid mesogenic units linked by a flexible chain were synthesized (Fig. 1a). The dimeric mixtures were prepared by mixing the dimers with biphenyl mesogenic units (BPFOn)^10^ and those with triphenyl mesogenic units (TPFOn). Moreover, a series of rodlike LCs (TTPFs) comprising a terphenyl rigid core and two flexible chains with variable lengths were also achieved (Fig. 1b). Several analogs of TTPF for specific experimental requirements are listed in Fig. 1c–e. Two chiral dopants with high helical twisting power (HTP) values, BDH1281 and R5011 (Supplementary Fig. S33), were used to offer high chiral forces for forming the DTC structures. The BP temperature ranges of all the samples capillary filled into 20.0 μm-thick cells without alignment treatment were determined using polarizing optical microscopy (POM) with a cooling rate of 0.5 °C/min and corrected via differential scanning calorimetry (DSC) and the temperature dependence of the relative dielectric constant (*ε*_r_–*T*). The results are listed in Table 1.

**Table 1:** Compositions and phase-transition temperatures of the studied samples.

	Dimeric LC wt %	Rodlike LC wt %	Chiral dopant wt %		Transition temperature °C		
Sample No.	BPFOn/TPFOn	TTPF/TTP/DITPF/TP2FTF	R5011	BDH1281	Iso-BP^a^	BP-X^*b^	ΔT^c^
1	0.0/0.0	94.0/0.0/0.0/0.0	2.0	4.0	122.5	122.5	0.0
2	66.9/27.1	0.0/0.0/0.0/0.0	2.0	4.0	60.0	11.5	48.5
3	60.2/24.4	9.4/0.0/0.0/0.0	2.0	4.0	64.5	1.5	63.0
4	53.5/21.7	18.8/0.0/0.0/0.0	2.0	4.0	76.6	−9.7	86.3
5	46.8/19.0	28.2/0.0/0.0/0.0	2.0	4.0	85.7	−40.8	126.5
6	43.5/17.6	32.9/0.0/0.0/0.0	2.0	4.0	92.8	−39.6	132.4
7	40.1/16.3	37.6/0.0/0.0/0.0	2.0	4.0	96.0	66.5	29.5
8	43.5/17.6	18.8/0.0/14.1/0.0	2.0	4.0	72.8	−24.5	97.3
9	60.2/24.4	0.0/9.4/0.0/0.0	2.0	4.0	88.5	88.5	0.0
10	60.2/24.4	4.7/4.7/0.0/0.0	2.0	4.0	77.0	30.0	47.0
11	43.5/17.6	28.2/0.0/0.0/4.7	2.0	4.0	87.3	−40.5	127.8
12	43.5/17.6	23.5/0.0/0.0/9.4	2.0	4.0	80.6	−40.9	121.5
13	43.5/17.6	18.8/0.0/0.0/14.1	2.0	4.0	75.7	−41.3	117.0
14	39.3/21.8	14.1/0.0/0.0/18.8	2.0	4.0	83.5	−38.8	122.3

^a^Iso-BP is the phase transition temperature from an isotropic liquid to a BP.

^b^BP–X* is the phase transition temperature from a BP to an unidentified phase.

^c^ΔT is the temperature range of a BP.

### Phase parameters and performance of the material systems

**Fig. 2:**
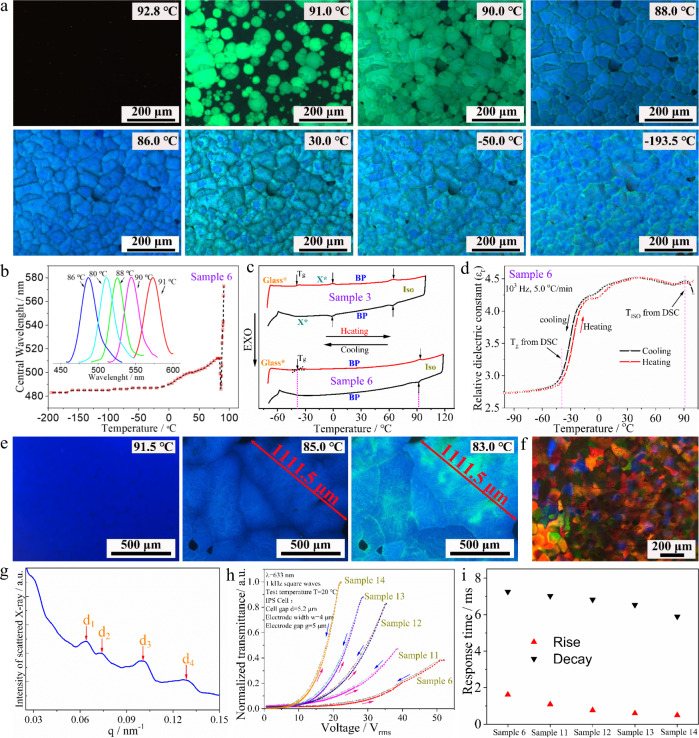
Characterization of BPs using POM, fiber optic spectrometer, DSC, impedance analysis, SAXS, and electro-optical testing. **a** POM textures of Sample 6 at different temperatures. **b** Temperature-dependent Bragg reflection of Sample 6. **c** DSC curves of Samples 3 and 6, phase transition points are marked via black arrows; corresponding temperatures are marked via red dashed lines. **d** Temperature dependence of relative dielectric constant (*ε*_r_–*T*) of Sample 6, corresponding phase transition points from DSC measurement are marked via red dashed lines. **e** Large BP single crystals obtained via thermal treatment. **f** POM textures of Sample 8 for SAXS measurement. **g** Intensity of scattered X-rays in arbitrary units as a function of the magnitude of the scattering vector q (an average value of three measures), corresponding scattered peaks of spacings d_1_–d_4_ are marked via red arrows. **h** Voltage-dependent transmittance (V-T). **i** Electro-optical response curves of the BP samples.

As listed in Table 1, BP is absent in Sample 1, which is composed of 94.0 wt% TTPF, 2.0 wt% R5011, and 4.0 wt% BDH1281, because the rod-shaped configuration of TTPF is not beneficial for forming DTC structures^5^. In contrast, BPs with a temperature range of 48.5 °C are observed in Sample 2, composed of 66.9 wt% BPFOn, 27.1 wt% TPFOn, 2.0 wt% R5011, and 4.0 wt% BDH1281, in which the biaxial configuration and flexoelectricity of the dimers are beneficial for stabilizing the DTC structures^2,5^. Interestingly, comparing the results of Samples 3–6, the BP temperature range substantially increased from 63.0 °C to over 132.4 °C upon increasing the concentration of rodlike TTPF in the samples from 9.4 wt% to 32.9 wt%, respectively. Figure 2a illustrates the optical textures of Sample 6 at different temperatures. When the sample is cooled to 92.8 °C, BP textures emerge, and they remain present until −193.5 °C (a video of the optical texture variation upon cooling is shown in Supplementary Movie S1). The Bragg reflection spectra of the BPs in Sample 6 upon cooling were measured using a fibre optical spectrometer and can always be detected from ~91.0 °C to −193.5 °C (Fig. 2b). The central reflection wavelength of the BPs decreases from approximately 573 nm to 486 nm upon cooling from 91.0 °C to 86.0 °C, respectively; however, it returns to ~512 nm at 80.0 °C and then decreases slowly again to 483 nm upon cooling to −193.5 °C. The inset in Fig. 2b shows the characteristic reflection spectra of the BP at 91.0, 90.0, 88.0, 86.0, and 80.0 °C. An abrupt rebound occurred during the decrease in the central reflection wavelength when Sample 6 was cooled from 88.0 °C to 86.0 °C and then from 86.0 °C to 80.0 °C, while the color of BP platelets changed from green to blue and then from blue to green, respectively. A phase transition from BPII to BPI at ~86.0 °C can be confirmed via the DSC measurement data of Sample 6 from 70.0 to 110.0 °C (Supplementary Fig. S34). Moreover, the wide-angle X-ray diffraction (WAXD) spectrum of Sample 6 was measured from 100.0 to −190.0 °C (Supplementary Fig. S35) and confirmed that no precipitation of crystals occurred during the cooling procedure in the sample.

Sample 6 in a low temperature near a liquid nitrogen temperature still maintained a BP state; generally, it may have been vitrified to a glass state, in which the structure of the BP was simply frozen. To determine the glass-transition temperature (*T*_g_), the thermograms of Samples 3 and 6 were measured via DSC (Fig. 2c). The phase transition temperatures Iso-BP and BP–X* from DSC are in accordance with those from POM, but the glass-transition peak was discovered only in the heating process. To further affirm this *T*_g_, the *ε*_r_–*T* curves were obtained via an impedance analyzer (Fig. 2d). A distinct variation at apporoximately −40.0 °C is exhibited in the heating and cooling processes, which is in accordance with the *T*_g_ obtained from DSC. Therefore, it can be concluded that the BP state of Sample 6 was a real thermal equilibrium state from 92.8 to −39.6 °C. Subsequently, a continuous increase in TTPF concentration to 37.6 wt% results in abrupt suppression of the range in Sample 7. Thus, an appropriate concentration of TTPF can substantially stabilize the cubic nanostructures of the BPs.

Furthermore, large BP single crystals (Fig. 2e, the diameter of the maximum platelet exceeds 1100 μm) can be obtained from this system by only ~3 h of thermal treatment upon cooling the sample in a 20.0 μm-thick cell, with no initial surface treatment, from the isotropic phase to 91.5 °C at ~0.1 °C/min, then from 91.5 to 88.0 °C at ~0.02 °C/min and then from 88.0 °C to 83.0 °C at ~0.5 °C/min. Highly effective approaches for fabricating a large BP single crystal were presented by developing advanced equipment and excellent professional technologies in previous studies^22,23^. We believe that better performance would be obtained if our material system was combined with these good types of equipment and technologies. Large BP single crystals easily form in this system, which is highly conducive to potential application in photonics fields^21^.

**Fig. 3:**
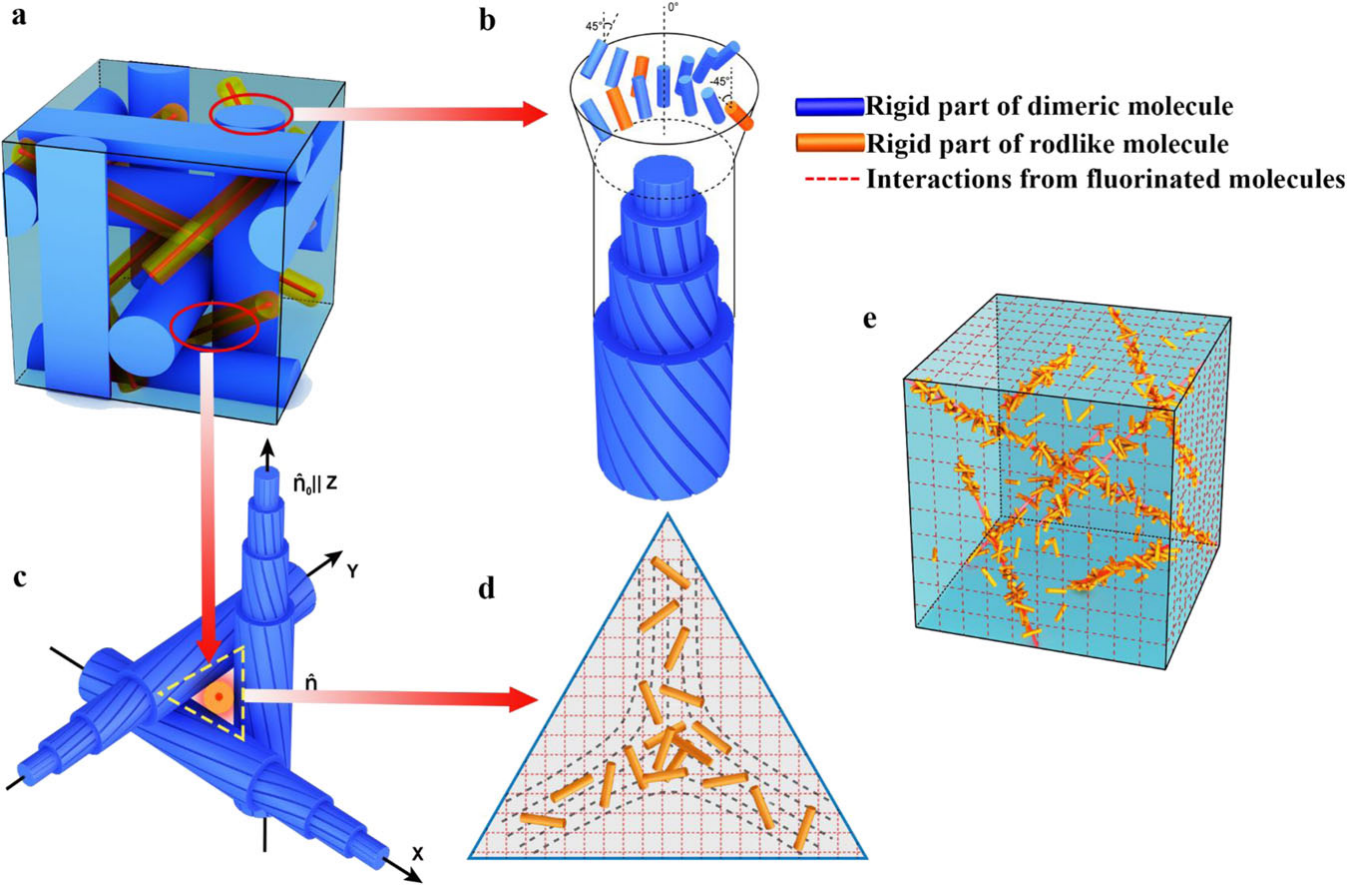
Schematic illustration of stabilizing BP by molecular synergistic self-assembly. **a** The DTC structures (O^8+^ structure) and the disclination lines (O^8−^ structure) in BP I. **b** The molecular arrangement of the DTCs in BPLCs. **c** A disclination formed by three crossed DTCs. **d** The defect region filled by rodlike molecules. **e** Defect lattices (O^8−^) stabilized by rodlike molecules.

In prior work, the relationship between the elastic constant (the values of our system are listed in the Supplementary Information) and molecular configuration was used by researchers to explain the effect of additives, such as bent-core molecules, in widening the BP temperature range. However, the underlying mechanism of the rodlike additive in widening the BP temperature range is very different from that of the bent-core additive. Therefore, a small-angle X-ray scattering (SAXS) technique was further employed to explore this mechanism via Sample 8 comprising DITPF (Fig. 1d), which is an iodine-functionalized rodlike mesogenic analog of TTPF. Sample 8 is composed of 43.5 wt% BPFOn, 17.6 wt% TPFOn, 18.8 wt% TTPF, 14.1 wt% DITPF, 2.0 wt% R5011, and 4.0 wt% BDH 1281, which also exhibits a wide temperature range of BPs, as listed in Table 1. The SAXS measurements were performed on a Xenocs Xeuss 2.0 SAXS instrument, in which the camera length was 2.4947 m and the X-ray wavelength was 0.1542 nm, when Sample 8 was sandwiched between two polyimide film substrates with a 300 μm-thick spacer. The POM textures of Sample 8 for SAXS measurement in the BP state are shown in Fig. 2f, and the diffraction data of Sample 8 in this state were obtained. In contrast, no diffraction signal is observed in Sample 8 at the isotropic phase or in Sample 6 containing TTPF without iodine substituents. Figure 3g shows the intensity of scattered X-rays in arbitrary units as a function of the magnitude of the scattering vector q, where the diffraction peaks were *q*_1_ = 0.065, *q*_2_ = 0.075, *q*_3_ = 0.100 and *q*_4_ = 0.130 nm^−1^. Based on the *q* values and Woolf-Bragg’s equation (*d* = 2π/q)^24^, the d spacing can be calculated as follows: *d*_1_ = 96.7, *d*_2_ = 83.8, *d*_3_ = 62.8 and *d*_4_ = 48.3 nm. The ratio of *d*_1_:*d*_2_:*d*_3_:*d*_4_ is 1:0.87:0.65:0.50. A BCC lattice of BP I belongs to the space group symmetry *I*4_1_32 (O^8^), and the lattice symmetries of disclinations are the O^8−^ structure^3^. For O^8−^ lattices, the theoretical spacing ratio is *d*_211_:*d*_220_:*d*_321_:*d*_422_ = 1:0.87:0.66:0.50. Comparing the theoretical calculations with our experimental results, the distribution of DITPF in BP is consistent with an O^8−^ structure; that is, there is some tendency of DITPF to gather into the regions near the disclinations in BP I^25^.

Furthermore, as listed in Table 1, Sample 9 comprises 9.4 wt% TTP without a fluorine atom attaching to the aromatic rings; and Sample 10, 4.7 wt% TTPF and 4.7 wt% TTP. They exhibit BP temperature ranges of 0.0 °C and 47.0 °C, respectively, which are both distinctly narrower than that of Sample 3 comprising 9.4 wt% TTPF (63.0 °C). In fluorinated molecules, the excellent stability of the C–F bond as well as the small size and low polarizability of the fluorine give rise to very low intermolecular dispersion interactions, which result in subtle modifications regarding fundamental chemical and physical properties of the liquid crystals, such as the melting point and transition temperatures, as well as dielectric, optical and viscoelastic properties^26^. Therefore, TTPF exhibits a more extensive LC phase range and better intermiscibility than those of TTP, and their transition temperatures are shown in Supplementary Figs. S29 and S30, respectively. Notably, the weak interactions^26^ from fluorinated molecules may play a beneficial effect in stabilizing the cubic nanostructures of the BPs.

The electro-optic performances of the BP materials were investigated in a non-oriented IPS cell with a cell gap of 5.2 μm, indium tin oxide electrode width of 4 μm and an electrode gap of 5 μm. The voltage-dependent transmittance (VT) curves and the electro-optical response times of the BP samples were measured with a He–Ne laser *λ* = 633 nm at 20 °C. The VT curve of Sample 6 shows that the increase in the transmittance is very slight when *V*_rms_ < 30 V (Fig. 2h). To improve the performance, an analog of TTPF with three terminal groups of fluoro substituents named TP2FTF (Fig. 1e) was used. Compared with Sample 6, 4.7 wt%, 9.4 wt% and 14.1 wt% TTPFs were replaced by TP2FTF in Samples 11–13, respectively. The electro-optical performances of the samples improve observably upon increasing the concentration of TP2FTF; however, the BP range decreases distinctly because of the decrease in the clearing point. Therefore, the proportion of TPFOn with a high clearing point was simultaneously increased in Sample 14 when the concentration of TP2FTF was further increased to 18.8 wt%. Sample 14 exhibits a stable BP state from −38.8 °C to 83.5 °C, and the voltage is ~20 V_rms_ at the maximum transmittance, which is less than half of that of Sample 6 (approximately 50 V_rms_), as shown in the V-T curve of Fig. 2h. Comparing the electro-optical response times of Sample 6 with those of Samples 11–14 shows that the rise time decreases from ~1.62 ms to 0.493 ms upon the increase in TP2FTF with large polarity (Fig. 2i). However, the decay time slightly decreases at ~6–7 ms because of the high viscosity of the material system. From the above, we can believe that the system will be endowed with better electro-optical performance when a more appropriate combination of materials is constructed in the future.

## Discussion

It is well known that the BCC lattices of BP I are packed by DTCs (O^8+^ structure) and topological defects (O^8−^ structure)^3^, and the existence of high energy defects (Fig. 3a, e) can destabilize BP cubic superstructures^2^. The stability of BPs is usually dependent on a well-arranged DTC structure and suppression of defect free energy^12^. Here, the free energy was suppressed by the uniaxial mesogens filling the defect regions of the cubic lattices (Fig. 3c, d). Then, the biaxial dimers and uniaxial mesogenic molecules together construct the stable DTC structure (Fig. 3b). Moreover, the weak interactions between the fluorinated LC molecules are indispensable for forming ultrastable BPs.

Effectively filling the defect cores and reducing the total free energy is the most effective strategy for stabilizing BPs^1,7^. The theoretical understanding of BPs is based on the Landau–de Gennes theory^3^, and the free energy density is written as

1 $$f_{{\mathrm{full}}} =	 \, f_{{\mathrm{grad}}} + f_{{\mathrm{bulk}}}\\ =	 \, \frac{1}{4}K_1\left[ {\left( {\nabla \times {\mathrm{Q}}} \right)_{ij} + 2q_0Q_{ij}} \right]^2 + \frac{1}{4}K_0\left[ {\left( {\nabla \times {\mathrm{Q}}} \right)_i} \right]^2\\ 	+ c\,{\mathrm{Tr}}\,{\mathrm{Q}}^2 - \sqrt 6 b\,{\mathrm{Tr}}\,{\mathrm{Q}}^3 + a\left( {{\mathrm{Tr}}\,{\mathrm{Q}}^2} \right)^2$$

The first two terms represent the gradient free energy density *f*_grad_, and the last three terms represent the bulk free energy density *f*_bulk_; these five terms constitute the full free energy density *f*_full_. To assess the stability of a BP with filled defects, it is necessary to compare the full free energy *f*_full_ of a BP with that of a guest component filling the defects with the Ch phase in the same state. The free energy density profile *f(r)* can be obtained from the calculated order-parameter profile of BP I at a given temperature, and to minimize the free energy, the region of volume fraction *ϕ* with a higher free energy density *f*_h_ in the BP I should be replaced by the guest component with a lower free energy density * f*_l_ of the same volume fraction. A relevant inference was achieved by Fukuda^27^, and the *f*_full_ of a BPLC and a ChLC with a guest component filling the defects was given as *F*_BP_ and *F*_Ch,_ respectively.

2 $$F_{BP} = {\int}_{{\mathrm{{\Omega}}}_{{\mathrm{full}}} - {\mathrm{{\Omega}}}_{{\mathrm{guest}}}} {drf\left( r \right) + \;{\mathrm{{\Omega}}}_{{\mathrm{full}}}(\phi f_{{\mathrm{guest}}} + \;\sigma s)} ,$$

3 $$F_{{\mathrm{Ch}}} = {\mathrm{{\Omega}}}_{{\mathrm{full}}}\left\{ {\left( {1 - \phi } \right)f_{{\mathrm{Ch}}} + \phi f_{{\mathrm{guest}}}} \right\},$$

where *Ω*_full_ and *Ω*_guest_ represent the full region and the one replaced by the guest component, respectively; *ϕ* is a guest component of the volume fraction; *f*_guest_ is the free energy density of the guest component; *σ* is the interfacial energy; and *s* is the area of the interface per unit volume.

From Eqs. (2) and (3), the free energy difference per unit volume between BP and Ch in the same state is given by

4 $$F_{{\mathrm{BP}}} - F_{{\mathrm{Ch}}} = {\int}_{{\mathrm{{\Omega}}}_{{\mathrm{full}}} - {\mathrm{{\Omega}}}_{{\mathrm{guest}}}} {drf\left( r \right) + {\mathrm{{\Omega}}}_{{\mathrm{full}}}\left\{ { - \left( {1 - \phi } \right)f_{{\mathrm{Ch}}} + \sigma s} \right\}} .$$

Here, the free-energy difference per unit volume primarily depends on the guest component of volume fraction *ϕ* and the interfacial energy per unit volume *σ*. Fukuda computationally determined that the temperature range of stable BP I can be larger than 60 °C by introducing a guest component of volume fraction less than 10% when *σ* is 10^−5^ J m^−2^. Kikuchi et al. broadened the temperature range of a stable BP I to larger than 60 °C by introducing ~8 wt% polymer networks^1^, which conforms to the above theory.

The above results show that choosing suitable materials, which lead to low interfacial energy with the LC molecules in the DTCs, to fill the defects in the cubic lattice and reduce the total free energy is crucial for the stability of the BPs^27^. In the case of defects filled by polymers or nanoparticles, the free energy gain due to the replacement of the defect core region is sufficiently large to overcome the free energy loss from interfaces, so the stable range of BPs is increased^1,7^. In our system, the interfacial energy *σ* between the dimeric LCs and rodlike LCs is undoubtedly lower than that between the LCs and polymer networks in PSBP. The proportion of the rodlike LCs as the guest component exceeds 30 wt% in our system, and the volume fraction *ϕ* exceeds 15% even if half of the rodlike LCs remain in the defect region. According to the above theory, there is a tremendous gain in the temperature range of a BP. Therefore, a BP with a temperature range of over 100 °C can be obtained successfully in our system.

**Fig. 4:**
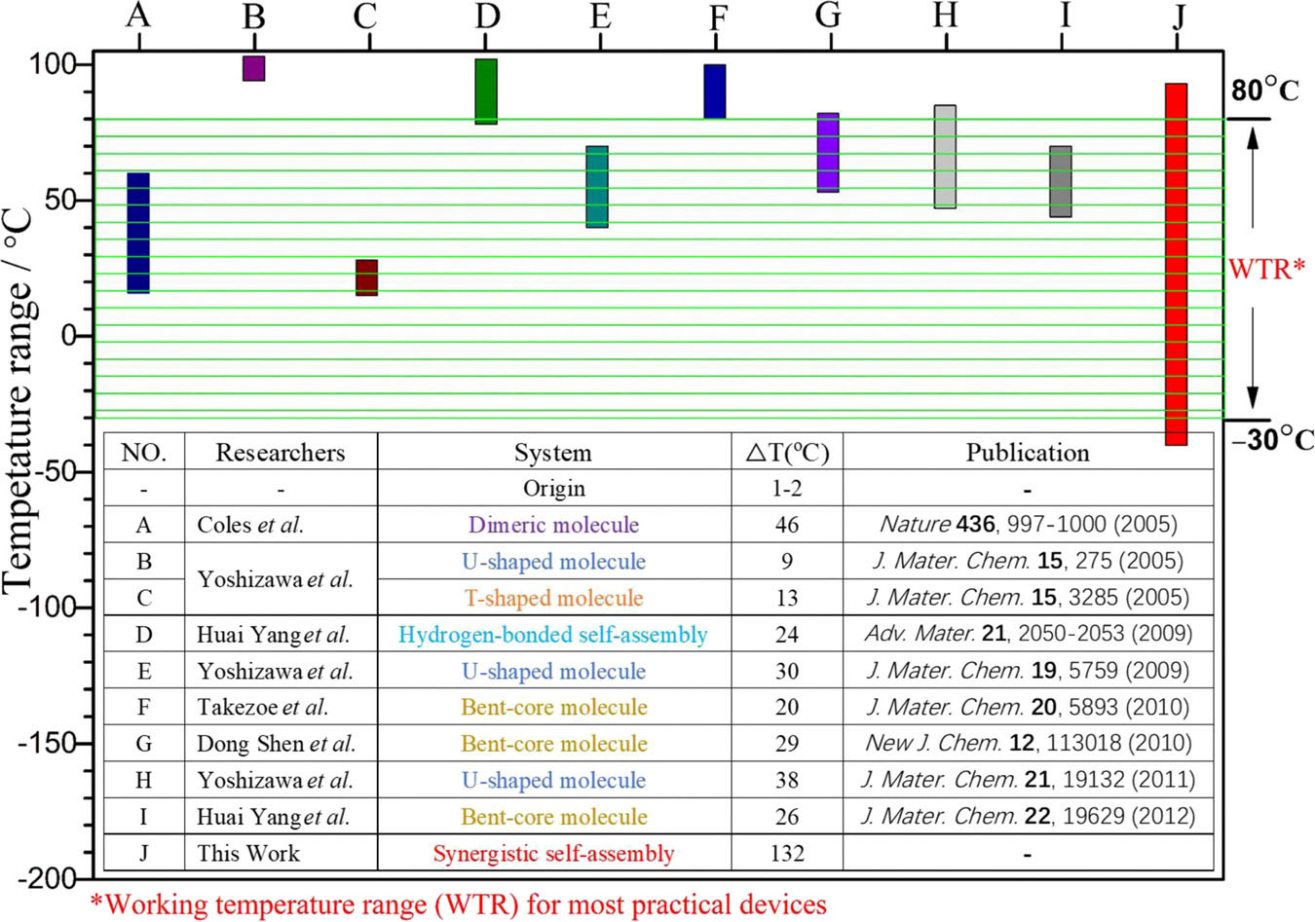
Development course of the temperature range of low molecular weight BPLCs. The column diagram indicates the temperature range of low molecular weight BPLCs obtained by different means, and the inset table shows the development course summary of low molecular weight BPLCs.

Molecular engineering aims to guide the assembly of atomic and molecular constituents into organized complex artificial materials with nanometre-sized precision and advanced functionalities^28^. Establishing a unique system for facilitating the formation of BPs via molecular design has become effective exploitation of molecular engineering, and the temperature range of BPs has been successfully broadened from a few degrees centigrade to tens of degrees centigrade. Here, the development course of low molecular weight BPLCs is summarized in Fig. 4. To date, all the methods to broaden the temperature range of BPs only by designing molecules to facilitate the formation of DTC structures are insufficient for obtaining a BP system covering the working temperature range of practical LC devices, as shown in Fig. 4. Our approach of engineering the synergistic self-assembly of molecules with distinct configurations to stabilize BPs can overcome this bottleneck, and it has been preliminarily proved to be effective for other systems in addition to the dimeric molecules system. For example, the effect of TTPF on broadening the BP temperature range also works with the commercial SLC-X previously reported^12^, as shown in Supplementary Fig. S36. We believe that better combinations of materials will be constructed in the future, which can be tailored with targeted properties satisfying different application requirements.

In this work, we successfully developed an ultrastable BP system from the molecular synergistic self-assembly of molecules with distinct configurations, which can be endowed with desirable properties by different material combinations for application requirements. Based on these results, we think this molecular synergistic self-assembly of a multi-component system may also be an extremely attractive approach to developing other multifunctional soft nanomaterials.

## Methods

### Materials

The chiral dopant R5011 is a commercial product (HCCH), and other materials are synthesized in our lab. The syntheses were performed through the Suzuki coupling reaction and Williamson etherification. The detailed syntheses and characterizations of the materials are shown in the Supporting Information. All chemicals and solvents were purchased from commercial suppliers and used without further purification.

### Measurements

The optical textures of the samples sandwiched between two glass substrates that contained 20.0 µm-thick polyester spacers were measured using a polarizing optical microscope (Carl Zeiss, AxioVision SE64) equipped with a hot stage with an accuracy of 0.1 °C (Linkam LTS420). Reflection spectra were taken using a fibre spectrometer (Avantes, AvaSpec-ULS2048) with a white light source.

The phase transition temperatures were investigated via DSC (Perkin Elmer Pyris 6) at a rate of 5.0 °C/min. The dielectric properties were characterized via the resonance method with an impedance analyzer (HP 4294A, Agilent Technology, USA) according to IEEE standards. The EO performances were studied by applying a 1 kHz AC electric field across the sample, and the response time was detected using a photoelectric converter connected to an oscilloscope.

Sample 8 used for SAXS measurements was composed of 43.5 wt% BPFOn, 17.6 wt% TPFOn, 18.8 wt% TTPF, 14.1 wt% DITPF, 2.0 wt% R5011, and 4.0 wt% BDH 1281 and then sandwiched between 300 μm-thick substrates of polyimide films. To obtain a better optical texture (Fig. 2c), the sample was first cooled from 80.0 to 65.0 °C at 0.05 °C/min and then from 65.0 °C to room temperature at 0.5 °C/min. The SAXS measurements were performed with a Xeuss 2.0. The camera length was 2.4947 m, and the X-ray wavelength was 0.1542 nm. The samples were exposed to X-ray radiation for 30 min three times, and then the SAXS measurement was averaged to obtain the SAXS data. The WAXD measurements were performed at Seuss 2.0 SAXS instrument of Xenocs.

Phase state and corresponding transition temperatures of LCs, as well as disclination line distance changes with the temperature to HTP of the used chiral dopants, were measured by polarizing optical microscope (Carl Zeiss, AxioVision SE64) equipped with a hot stage with an accuracy of 0.1 °C (Linkam LTS420). The phase transition temperatures were investigated by DSC (Perkin Elmer Pyris 6) at a rate of 10.0 °C/min. The elastic constants (*K*) of the system were obtained by the measurements through the use of Instec ALCT instrument.

## Supplementary information

Supplementary Information

Description of Additional Supplementary File

Supplementary Movie

## Data Availability

Data supporting the findings of this study are available within the article (and its Supplementary Information files) and from the corresponding author upon reasonable request.
