# Supplementary material for: Ultrastable liquid crystalline blue phase from molecular synergistic self-assembly
Source: Nat Commun. 2021 Mar 4;12:1440. doi: 10.1038/s41467-021-21564-y (PMC7933424; doi:10.1038/s41467-021-21564-y)
Supplement: Supplementary file 1 — Supplementary Information [file 41467_2021_21564_MOESM1_ESM.docx]

Supporting Information

**Ultrastable Liquid Crystalline Blue Phase from Molecular Synergistic Self-Assembly**

*Wei Hu^a,b^, Ling Wang ^c^, Meng Wang^a^, Tingjun Zhong^a^,* *Qian Wang^a^, Lanying Zhang^a^, Feiwu Chen^b^, Kexuan Li^d^, Zongcheng Miao^d^, Dengke Yang^e^ and Huai Yang^a*^*

Supplementary Note 1

Syntheses and characterizations of materials

The syntheses were carried out through Suzuki coupling reaction[^1^](#_ENREF_1)^,^[^2^](#_ENREF_2) and Williamson etherification^[3](#_ENREF_3)^.

General/Typical Procedure of Suzuki coupling reaction: A commercial Pd catalytic agent (0.1% mol), aryl bromide (1.0 mmol), arylboronic acid (1.3 mmol), powdered Na_2_CO_3_ (2.0 mmol), and ethylene glycol monomethylethe/water = 4/1 (5.0 mL) was placed in a three-necked, round-bottomed flask equipped with a magnetic stirrer. The reaction was allowed to stir at 80.0 °C until the starting material disappeared (monitored by TLC). The mixture was then diluted with water (15 mL) and extracted with CH_2_Cl_2_. The combined organic phase was dried over anhydrous Na_2_SO_4_ and the solvent was evaporated under vacuum. The product was further purified by chromatography.

General/Typical Procedure of Williamson etherification reaction: A mixture of aryl phenol (2.50 mmol), anhydrous potassium carbonate (4.78 mmol) and dibromo alkane (1.27 mmol, ) in dry dimethylformamide (DMF) (20.0 mL) was placed in a three-necked, round-bottomed flask equipped with a magnetic stirrer. The reaction was allowed to stir at 80.0 °C until the starting material disappeared (monitored by TLC). Water (40.0 mL) was added and the mixture was allowed to cool to room temperature with stirring. The precipitate was filtered, dissolved in a large amount of chloroform and filtered. Ethanol was added to the filtrate and the precipitate was collected and dried under vacuum. The product was further purified by chromatography.

All products were characterized by NMR spectroscopy. ^1^H NMR (400 MHz) spectra and ^13^C NMR (126 MHz) spectra were recorded on a Bruker 500 spectrometer. Chemical shifts are in δ units (ppm) with the residual solvent peak or TMS as the internal standard. The coupling constant (J) is reported in Hertz (Hz). NMR splitting patterns are designated as follows: s, singlet; d, doublet; t, triplet; q, quartet; and m, multiplet.

Syntheses and characterizations of TPFOn

Supplementary Figure 1 | Synthesis routes for BPFOn. The BPFOn are synthesized via one step Suzuki cross coupling reaction and one step Williamson etherification.

Supplementary Figure 2 | Chemical stucture of BPFO. The characterization data as follows: ^1^H NMR (400 MHz, Chloroform-*d*): δ= 7.45 (m, J = 6.9, 5.4, 1.7 Hz, 2H), 7.29 – 7.23 (m, 1H), 7.17 – 7.04 (m, 2H), 6.74 – 6.59 (m, 2H), 4.98 (s, 1H). Elemental analysis: Calcd. for C_12_H_8_F_2_O: C 69.90%, H 3.91%; found: C 69.93%, H 3.89%.

Supplementary Figure 3 | Chemical stucture of BPFO7. The characterization data as follows: ^1^H NMR (400 MHz, Chloroform-*d*): δ= 7.46 (m, J = 8.9, 5.3, 1.6 Hz, 4H), 7.29 (t, J = 8.8 Hz, 2H), 7.10 (t, J = 8.7 Hz, 4H), 6.80 – 6.65 (m, 4H), 3.99 (t, J = 6.4 Hz, 4H), 1.83 (m, J = 6.5 Hz, 4H), 1.50 (d, J = 6.5 Hz, 6H). Elemental analysis: Calcd. for C_31_H_28_F_4_O_2_: C 73.22%, H 5.55%; found: C 73.15%, H 5.57%.

Supplementary Figure 4 | Chemical stucture of BPFO9. The characterization data as follows: ^1^H NMR (400 MHz, Chloroform-*d*): δ= 7.46 (m, J = 8.6, 6.0, 1.6 Hz, 4H), 7.28 (t, J = 8.9 Hz, 2H), 7.10 (t, J = 8.5 Hz, 4H), 6.86 – 6.60 (m, 4H), 3.97 (t, J = 6.5 Hz, 4H), 1.80 (m, J = 6.8 Hz, 4H), 1.52 – 1.29 (m, 10H). Elemental analysis: Calcd. for C_33_H_32_F_4_O_2_: C 73.86%, H 6.01%; found: C 73.90%, H 6.05%.

Supplementary Figure 5 | Chemical stucture of BPFO11. The characterization data as follows: ^1^H NMR (400 MHz, Chloroform-*d*): δ= 7.46 (m, J = 6.9, 5.4, 1.6 Hz, 4H), 7.30 (d, J = 8.9 Hz, 2H), 7.16 – 7.00 (m, 4H), 6.80 – 6.65 (m, 4H), 3.97 (t, J = 6.5 Hz, 4H), 1.79 (p, J = 14.8, 6.5 Hz, 4H), 1.51 – 1.42 (m, 4H), 1.38 – 1.29 (m, 10H). Elemental analysis: Calcd. for C_35_H_36_F_4_O_2_: C 74.45%, H 6.43%; found: C74.43%, H 6.45%.

Syntheses and characterizations of TPFOn

Supplementary Figure 6 | Synthesis routes for TPFOn. The TPFOn are synthesized via two steps Suzuki cross coupling reaction and one step Williamson etherification.

Supplementary Figure 7 | Chemical stucture of TPFO. The characterization data as follows:^1^H NMR (400 MHz, Chloroform-d): δ= 7.54 – 7.47 (m, 1H), 7.43 – 7.38 (m, 2H), 7.34 (d, J = 12.2 Hz, 1H), 7.25 – 7.17 (m, 2H), 6.99 – 6.88 (m, 2H), 5.07 (s, 1H). Elemental analysis: Calcd. for C_18_H_10_F_4_O: C 67.93%, H 3.17%; found: C 67.90%, H 3.20%.

Supplementary Figure 8 | Chemical stucture of TPFO7. The characterization data as follows: ^1^H NMR (400 MHz, Chloroform*-d*): δ= 7.57 – 7.50 (m, 2H), 7.44 – 7.32 (m, 6H), 7.22 (m, J = 8.8, 6.5, 1.2 Hz, 4H), 7.05 – 6.93 (m, 4H), 4.03 (t, J = 6.4 Hz, 2H), 1.93 – 1.78 (m, 4H), 1.60 – 1.55 (m, 2H), 1.54 – 1.47 (m, 4H). ^13^C NMR (126 MHz, Chloroform-d) δ 160.81, 159.48, 158.83, 152.16, 150.18, 143.26, 140.33, 138.32, 133.58, 131.50, 131.27, 130.33, 128.02, 123.93, 122.65, 115.02, 114.24, 113.02, 68.11, 29.53, 26.02. Elemental analysis: Calcd. for C_43_H_32_F_8_O_2_: C 70.49%, H 4.40%; found: C 70.50%, H 4.41%.

Supplementary Figure 9 | Chemical stucture of TPFO9. The characterization data as follows: ^1^H NMR (400 MHz, Chloroform*-d*): δ= 7.62 – 7.47 (m, 4H), 7.47 – 7.30 (m, 6H), 7.22 (m, J = 8.9, 6.6, 1.2 Hz, 4H), 7.06 – 6.89 (m, 4H), 4.01 (t, J = 6.5 Hz, 4H), 1.92 – 1.76 (m, 4H), 1.54 – 1.33 (m, 10H). ^13^C NMR (126 MHz, Chloroform-d) δ 160.81, 159.49, 158.83, 152.22, 150.18, 143.27, 140.39, 138.32, 133.58, 131.50, 131.26, 130.34, 128.01, 123.99, 122.67, 115.02, 114.34, 113.09, 68.15, 29.45, 29.26, 26.03. Elemental analysis: Calcd. for C_45_H_36_F_8_O_2_: C 71.05%, H 4.77%; found: C 71.10%, H 4.75%.

Supplementary Figure 10 | Chemical stucture of TPFO11. The characterization data as follows: ^1^H NMR (400 MHz, Chloroform*-d*): δ= 7.62 – 7.47 (m, 4H), 7.47 – 7.30 (m, 6H), 7.22 (m, J = 8.9, 6.6, 1.2 Hz, 4H), 7.06 – 6.89 (m, 4H), 4.01 (t, J = 6.5 Hz, 4H), 1.92 – 1.76 (m, 4H), 1.54 – 1.33 (m, 10H). ^13^C NMR (126 MHz, Chloroform-d) δ 160.81, 159.50, 158.83, 152.44, 150.18, 143.28, 140.39, 138.32, 133.58, 131.51, 131.23, 130.32, 128.01, 123.92, 122.65, 115.02, 114.24, 113.02, 68.18, 29.51, 29.37, 29.27, 26.05. Elemental analysis: Calcd. for C_47_H_40_F_8_O_2_: C 71.56%, H 5.11%; found: C 71.53%, H 5.10%.

Syntheses and characterizations of TTPF

Supplementary Figure 11 | Synthesis routes for TTPF. The TTPF are synthesized via two steps Suzuki cross coupling reaction.

Supplementary Figure 12 | Chemical stucture of TP2F3. The characterization data as follows: ^1^H NMR (400 MHz, Chloroform-*d*): δ= 7.59 – 7.45 (m, 5H), 7.44 – 7.33 (m, 2H), 7.30 – 7.24 (m, 4H), 2.80 – 2.53 (m, 4H), 1.70 (q, J = 7.5 Hz, 2H), 1.29 (m, J = 7.7, 1.4 Hz, 3H), 1.07 – 0.91 (m, 3H). Elemental analysis: Calcd. for C_23_H_23_F: C 86.75%, H 7.28%; found: C 86.70%, H 7.25%.

Supplementary Figure 13 | Chemical stucture of TP2F5. The characterization data as follows: ^1^H NMR (400 MHz, Chloroform-*d*): δ= 7.51 (m, J = 15.2, 10.8, 7.5 Hz, 5H), 7.44 – 7.33 (m, 2H), 7.30 – 7.23 (m, 4H), 2.68 (m, J = 16.0, 7.8 Hz, 4H), 1.67 (p, J = 7.5 Hz, 2H), 1.41 – 1.20 (m, 7H), 1.02 – 0.82 (m, 3H). Elemental analysis: Calcd. for C_25_H_27_F: C 86.66%, H 7.85%; found: C 86.60%, H 7.91%.

Supplementary Figure 14 | Chemical stucture of TP3F5. The characterization data as follows: ^1^H NMR (400 MHz, Chloroform-*d*): δ= 7.56–7.46 (m, 5H), 7.45–7.35 (m, 2H), 7.28–7.23 (m, 4H), 2.65 (q, J = 7.2 Hz, 4H), 1.67 (m, 4H), 1.45–1.30 (m, 4H), 1.06– 0.83 (m, 6H). Elemental analysis: Calcd. for C_26_H_29_F: C 86.62%, H 8.11%; found: C 86.60%, H 8.08%.

Syntheses and characterizations of TTP

Supplementary Figure 15 | Synthesis routes for TTP. The TTP are synthesized via two steps Suzuki cross coupling reaction.

Supplementary Figure 16 | Chemical stucture of TP23. The characterization data as follows: ^1^H NMR (500 MHz, Chloroform-*d*): δ= 7.62 (s, 4H), 7.55 – 7.50 (m, 4H), 7.30 – 7.23 (m, 4H), 2.81 – 2.55 (m, 4H), 1.68 (q, J = 7.7 Hz, 2H), 1.26 (m, J = 7.5, 1.5 Hz, 3H), 1.08 – 0.95 (m, 3H). Elemental analysis: Calcd. for C_23_H_24_: C 91.95%, H 8.05%; found: C 91.90%, H 8.10%.

Supplementary Figure 17 | Chemical stucture of TP25. The characterization data as follows: ^1^H NMR (500 MHz, Chloroform-*d*): δ= 7.64 (s, 4H), 7.59–7.52 (m, 4H), 7.31–7.25 (m, 4H), 2.76–2.61 (m, 4H), 1.66 (q, 4H), 1.41–1.33 (m, 7H), 1.29 (t, J = 7.6 Hz, 2H), 0.96 – 0.84 (m, 3H). Elemental analysis: Calcd. for C_25_H_28_: C 91.41%, H 8.59%; found: C 91.40%, H 8.60%.

Supplementary Figure 18 | Chemical stucture of TP35. The characterization data as follows: ^1^H NMR (500 MHz, Chloroform-*d*): δ= 7.65 (s, 4H), 7.56 – 7.50 (m, 4H), 7.30 – 7.23 (m, 4H), 2.62 (q, J = 7.5 Hz, 4H), 1.66 (m, J = 12.3, 6.7, 6.1 Hz, 4H), 1.46 – 1.32 (m, 4H), 1.09 – 0.88 (m, 6H). Elemental analysis: Calcd. for C_26_H_30_: C 91.17%, H 8.83%; found: C 91.20%, H 8.80%.

Syntheses and characterizations of DITPF

Supplementary Figure 19 | Synthesis routes for DITPF. The DITPF is synthesized via two steps Suzuki cross coupling reaction, one step Williamson etherification and one step iodination.

Supplementary Figure 20 | Chemical stucture of TP35. The characterization data as follows: ^1^H NMR (400 MHz, Chloroform-*d*): δ= 7.51 (q, J = 8.6, 8.2 Hz, 4H), 7.46 – 7.32 (m, 2H), 7.29 (d, J = 11.9 Hz, 3H), 6.92 (d, J = 8.2 Hz, 2H), 2.65 (t, J = 7.8 Hz, 2H), 1.67 (d, J = 7.6 Hz, 2H), 1.36 (m, J = 6.6, 4.0 Hz, 4H), 0.99 – 0.83 (m, 3H). Elemental analysis: Calcd. for C_23_H_23_FO: C 82.60%, H 6.93%; found: C82.65%, H 6.96%.

Supplementary Figure 21 | Chemical stucture of TP5FO6E. The characterization data as follows: ^1^H NMR (400 MHz, Chloroform-*d*): δ= 7.57 – 7.45 (m, 5H), 7.41 – 7.26 (m, 4H), 7.07 – 6.89 (m, 2H), 5.84 (m, J = 16.9, 10.2, 6.6 Hz, 1H), 5.11 – 4.87 (m, 2H), 4.02 (t, J = 6.5 Hz, 2H), 2.78 – 2.56 (m, 2H), 2.23 – 2.09 (m, 2H), 1.83 (m, J = 14.9, 6.5 Hz, 2H), 1.73 – 1.59 (m, 4H), 1.36 (h, J = 3.8, 3.3 Hz, 4H), 0.91 (m, J = 7.0, 5.9, 3.2 Hz, 3H). Elemental analysis: Calcd. for C_29_H_33_FO: C 83.61%, H 7.99%; found: C 83.65%, H 7.95%.

Supplementary Figure 22 | Chemical stucture of DITPF. The characterization data as follows: ^1^H NMR (400 MHz, Chloroform-*d*): δ= 7.58 – 7.44 (m, 5H), 7.40 –7.27 (m, 4H), 7.02 – 6.96 (m, 2H), 4.37 (m, J = 12.2, 8.9, 3.4 Hz, 1H), 4.16 – 3.96 (m, 3H), 3.72 (m, J = 11.9, 9.7 Hz, 1H), 2.65 (m, J = 8.8, 6.7 Hz, 2H), 2.23 – 1.72 (m, 4H), 1.70 – 1.53 (m, 4H), 1.40 – 1.30 (m, 4H), 0.96 – 0.88 (m, 3H). ^13^C NMR (126 MHz, Chloroform-d) δ159.48, 158.83, 143.26, 138.32, 131.50, 131.27, 130.33, 128.02, 123.93, 122.65, 115.02, 114.63, 113.86, 113.02, 68.11, 38.88, 29.46, 29.27, 29.18, 29.09, 26.02,14.06,12.59. Elemental analysis: Calcd. for C_29_H_33_FI_2_O: C 51.96%, H 4.96%; found: C 51.90%, H 4.93%.

Syntheses and characterizations of TP2FTF

Supplementary Figure 23 | Synthesis routes for TP2FTF. The TP2FTF is synthesized via two steps Suzuki cross coupling reaction.

Supplementary Figure 24 | Chemical stucture of TP2FTF. The characterization data as follows: ^1^H NMR (400 MHz, Chloroform-d): δ= 7.53–7.45 (m, 1H), 7.42–7.37 (m, 2H), 7.34 (d, 1H), 7.25–7.17 (m, 2H), 6.99–6.85 (m, 2H), 2.77–2.55 (m, 2H), 1.27 (m, 3H). Elemental analysis: Calcd. for C_20_H_14_F_4_ : C 72.72%, H 4.27%; found: C 72.70%, H 4.28%.

Syntheses and characterizations of BDH1281

Supplementary Figure 25 | Synthesis routes for BDH1281. The BDH1281 is synthesized via one step Williamson etherification, one step hydrolysis reaction and one step esterification reaction.

Supplementary Figure 26 | Chemical stucture of BDH1281. The characterization data as follows: ^1^H NMR (400 MHz, Chloroform-*d*): δ= 7.99 (q, J = 26.2, 8.9 Hz, 4H), 6.90 (t, J = 8.9 Hz, 4H), 5.50 – 5.34 (m, 2H), 5.03 (t, J = 5.0 Hz, 1H), 4.67 (d, J = 4.7 Hz, 1H), 4.18 – 3.89 (m, 8H), 1.80 (m, J = 12.5, 6.4, 5.4 Hz, 4H), 1.53 – 1.41 (m, 4H), 1.40 – 1.27 (m, 8H), 0.91 (t, J = 6.5 Hz, 6H). Elemental analysis: Calcd. for C_32_H_42_O_8_: C 69.29%, H 7.63%; found: C 69.25%, H 7.61%.

Supplementary Note 2

Characterizations of LCs

1. Dimeric LCs BPFOn


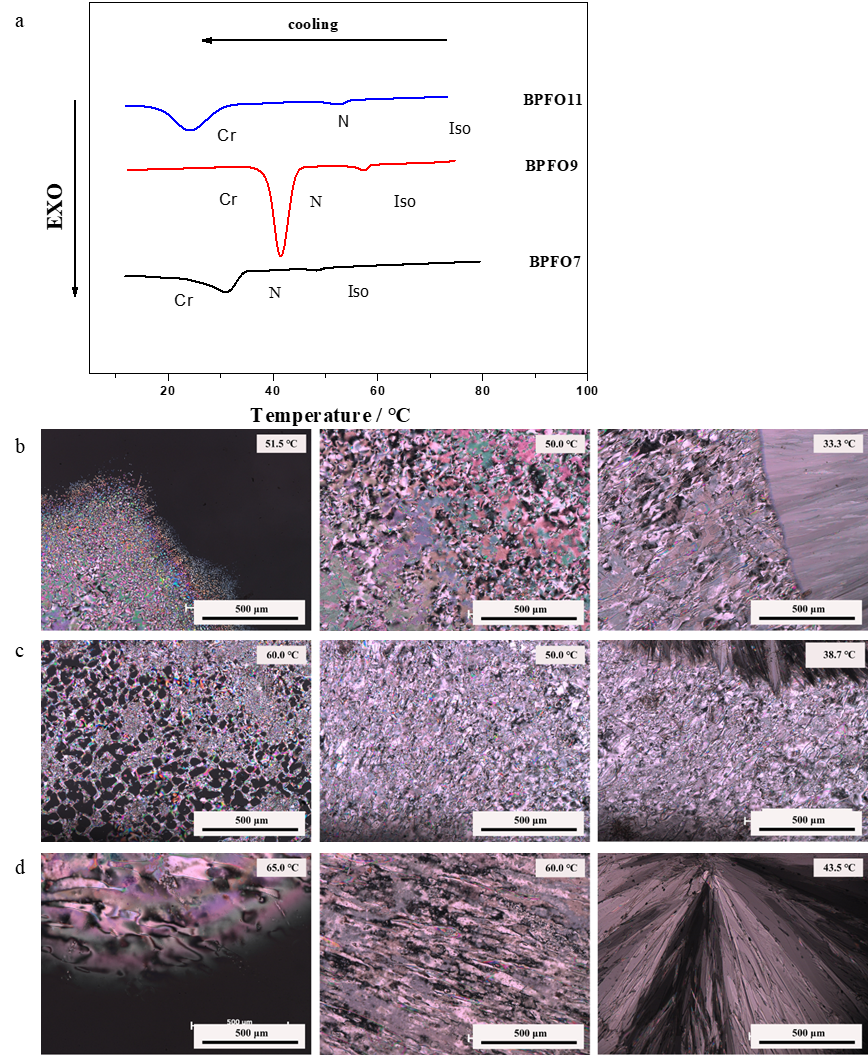


Supplementary Figure 27 | Characterizations of BPFOn. a, The DSC cooling curves of BPFOn at a rate of 10.0 °C/min. b, The optical textures of BPFO7 at different temperatures. c, The optical textures of BPFO9 at different temperatures. d, The optical textures of BPFO11 at different temperatures.

The transition temperatures of all the sample were determined at a cooling procedure by DSC and POM together, the characterization data are shown as follows:

BPFO7: I^a^ 51.5 °C N^b^ 33.3 °C Cr^c^;

BPFO9: I 60.0 °C N 38.7 °C Cr;

BPFO11: I 65.0 °C N 43.5 °C Cr;

^a^I is an isotropic phase, ^b^N is a nematic phase, ^c^Cr is a crystal phase.

It is especially pointed out that all BPFOn are monotropic liquid crystals.

1. Dimeric LCs TPFOn


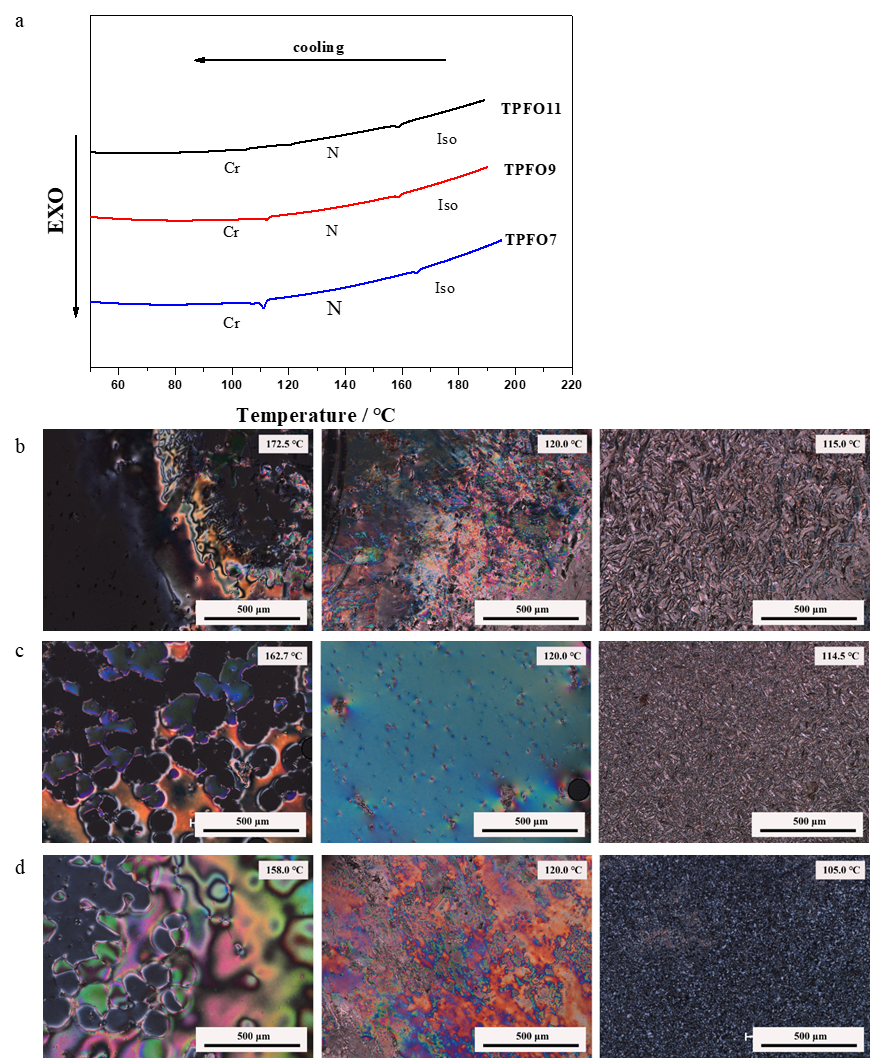


Supplementary Figure 28 | Characterizations of TPFOn. a, The DSC cooling curves of TPFOn at a rate of 10.0 °C/min. b, The optical textures of TPFO7 at different temperatures. c, The optical textures of TPFO9 at different temperatures. d, The optical textures of TPFO11 at different temperatures.

The transition temperatures of all the sample were determined at a cooling procedure by DSC and POM together, the characterization data are shown as follows:

TPFO7: I 172.5 °C N 115.0 °C Cr;

TPFO9: I 162.7 °C N 114.5 °C Cr;

TPFO11: I 158.0 °C N 105.0 °C Cr.

1. Rodlike LCs TTPF


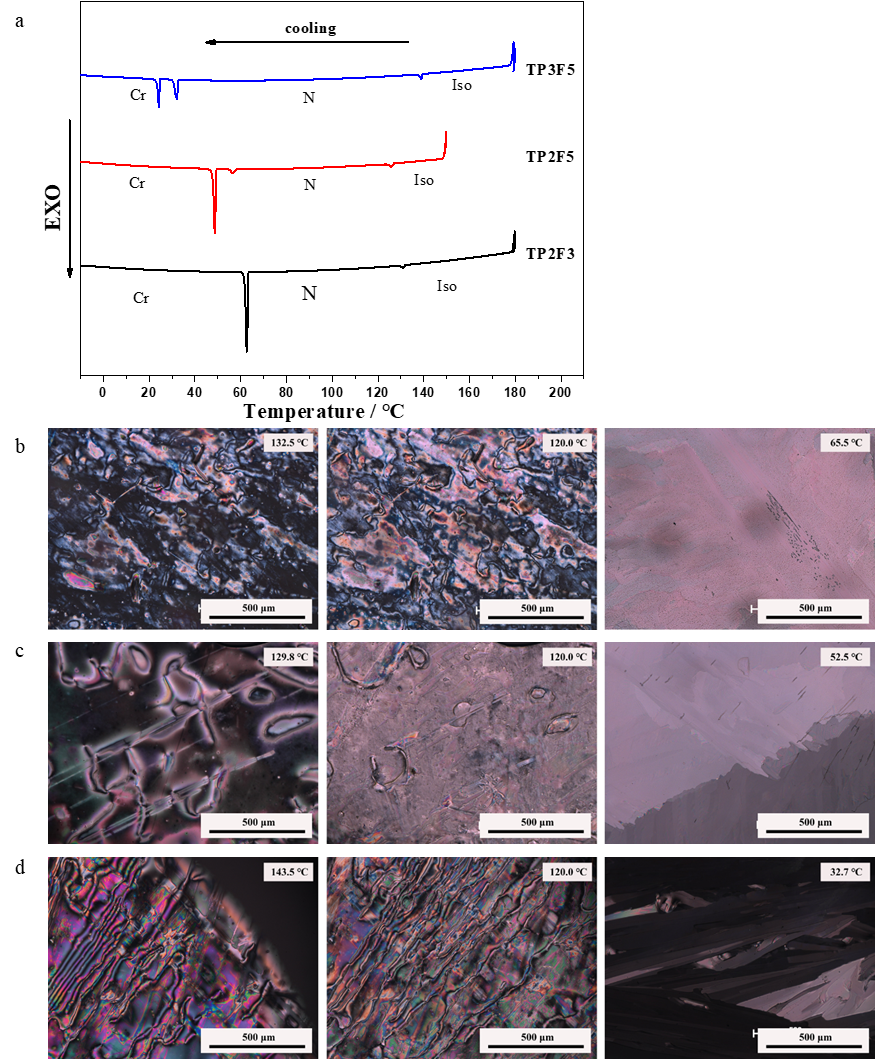


Supplementary Figure 29 | Characterizations of TTPF. a, The DSC cooling curves of TTPF at a rate of 10.0 °C/min. b, The optical textures of TP2F3 at different temperatures. c, The optical textures of TP2F5 at different temperatures. d, The optical textures of TP3F5 at different temperatures.

The transition temperatures of all the sample were determined at a cooling procedure by DSC and POM together, the characterization data are shown as follows:

TP2F3: I 132.5 °C N 65.5 °C Cr;

TP2F5: I 129.8 °C N 52.5 °C Cr;

TP3F5: I 143.5 °C N 32.7 °C Cr.

It is especially pointed out that the TP2F3 and TP2F5 have a crystal-crystal transition at 24.3 °C and 48.7 °C, respectively.

1. Rodlike LCs TTP


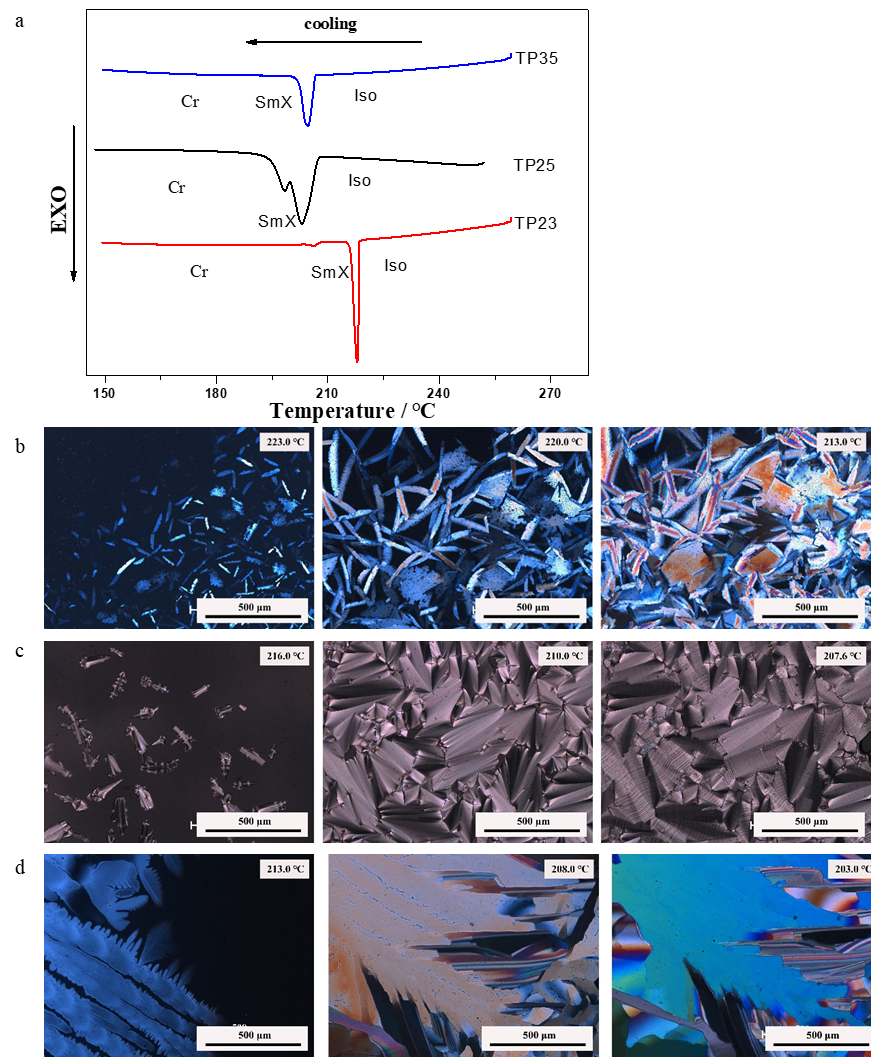


Supplementary Figure 30 | Characterizations of TTP. a, The DSC cooling curves of TTP at a rate of 10.0 °C/min. b, The optical textures of TP23 at different temperatures. c, The optical textures of TP25 at different temperatures. d, The optical textures of TP35 at different temperatures.

The transition temperatures of all the sample were determined at a cooling procedure by DSC and POM together, the characterization data are shown as follows:

TP23: I 223.0 °C SmX 213.0 °C Cr;

TP25: I 216.0 °C SmX 207.6 °C Cr;

TP35: I 213.0 °C SmX 203.0 °C Cr.

1. Rodlike LC DITPF and TP2FTF


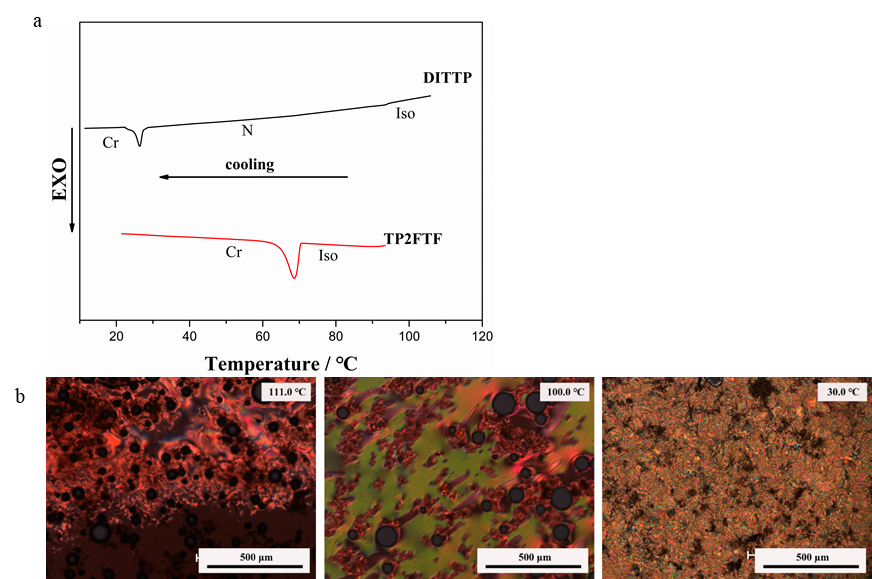


Supplementary Figure 31 | Characterizations of DITPF. a, The DSC cooling curves of DITPF and TP2FTF at a rate of 10.0 °C/min. b, The optical textures of DITPF at different temperatures.

The transition temperatures of DITPF and TP2FTF were determined at a cooling procedure by DSC and POM together, the characterization data are shown as follows:

DITPF: I 111.0 °C N 30.0 °C Cr

The clear point of TP2FTF is far lower than the crystallization temperature.

Supplementary Note 3

Characterizations of HTP of chiral dopants

The chiral dopant R5011 is a commercial product (HCCH), and the chiral dopant BDH1281 was synthesized in our lab. Their HTP were determined by a Grandjean-Cano wedge cell[^4^](#_ENREF_4). Two chiral dopants were added to a commercial nematic LC host SLC1717 (SLC) to obtain 1.0 wt% chiral nematic LCs (CLCs) mixture. Then the resulting mixture was capillary-filled into a wedge cell (EHC, KCRK-07, tan *θ* = 0.0196). Because the value of the pitch is fixed, and the alignment is also fixed, the cholesteric LC arranges itself as depicted in Supplementary Figure 32. This arrangement produces disclination lines between areas that contain a different number of layers. The disclination lines, known as Cano lines, of the CLCs in the wedge cell can be seen through a transmissive-mode polarizing optical microscope. The HTP values were obtained from β = 1/(pc) (a positive sign for right-handed helix and a negative sign for left-handed helix), where c is the concentration of the chiral dopant, p is the pitch length of the CLCs. The pitch length of 1.0 wt% chiral dopant in LC1717 was examined at different temperatures by the Grandjean–Cano wedge method with equation p = 2L tan *θ*, where L is the distance between the Cano lines and θ is the wedge angle of wedge cells (tan *θ* = 0.0196).


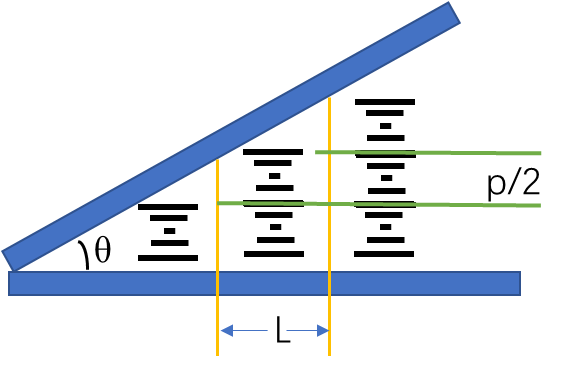


Supplementary Figure 32 | Schematic illustration of a Grandjean-Cano wedge cell for CLCs. Disclination lines are pointed out with arrows and the thickness change between two domains is marked as *p*/2.


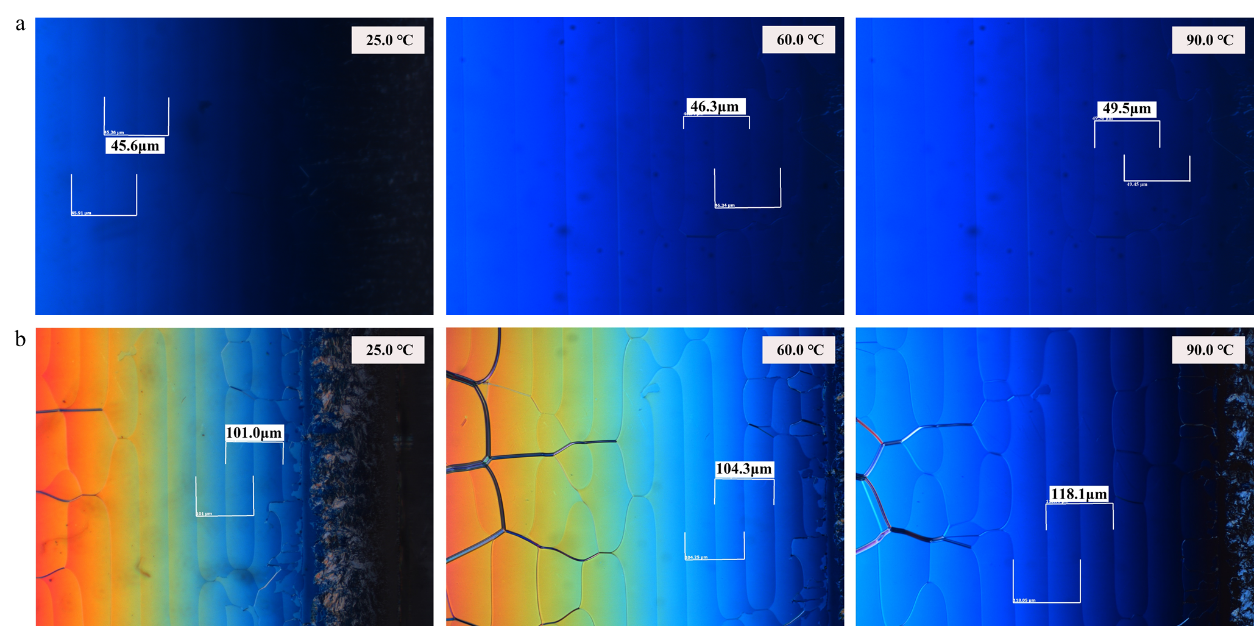


Supplementary Figure 33 | The HTP values of chiral dopants were determined in a wedge cell at different temperatures by observing through a crossed polarized transmissive mode optical microscopy. a, 1.0 wt% R5011 in SLC1717. b, 1.0 wt% BDH1281 in SLC1717.

The HTP values of R5011 in SLC1717

25.0 °C 2L = 45.6 μm, β = 1/(C* 2L* tan *θ*) = 111.9 μm^−1^;

60.0 °C 2L = 46.3 μm, β = 1/(C* 2L* tan *θ*) = 110.2 μm^−1^;

90.0 °C 2L = 49.5 μm, β = 1/(C* 2L* tan *θ*) = 103.1 μm^−1^.

The HTP values of BDH1281 in SLC1717

25.0 °C 2L = 101.0 μm, β = 1/(C* 2L* tan *θ*) = 50.5 μm^−1^;

60.0 °C 2L = 104.3 μm, β = 1/(C* 2L* tan *θ*) = 48.9 μm^−1^;

90.0 °C 2L = 118.1 μm, β = 1/(C* 2L* tan *θ*) = 43.2 μm^−1^.

The distance between two Cano lines expanded with the increased temperatures observed by a transmission-mode POM, which means that the HTP decreases with the increased temperatures.

Supplementary Note 4

Elastic constant measurement of the materials

In the prior work, the bent-shaped compound is found to be beneficial to the BP stability rather than the straight-shaped compound. Their study present that the stability of the BP is greatly enhanced when the bend elastic constant (*K_33_*) is smaller, and also that larger splay (*K*_11_) and twist (*K_22_*) elastic constants stabilize the BP. With the increase in the amount of the bent-core additive, *K_33_/K_11_* of the nematic host mixture becomes smaller, widening the BP temperature range of the resulting mixture between the host and the chiral dopant. As a contrast，the elastic constants of our system were obtained by the measurements through the use of Instec ALCT instrument.

FOA：*K*_11_=4.22, *K_33_=*4.58, *K_33_/K_11_*=1.09;

TTPF: *K*_11_=18.30, *K_33_=*34.03, *K_33_/K_11_*=1.86;

Host mixture of Sample 6: *K*_11_=8.71, *K_33_=*13.73, *K_33_/K_11_*=1.58.

The *K_33_/K_11_* of the nematic host mixture becomes larger with the addition of the rodlike LC molecules TTPF, which accord with the prior work about the relationship between the elastic constant and molecule configuration.

Supplementary Note 5

DSC measurement of Sample 6 from 70.0 °C to 110.0 °C





Supplementary Figure 34 | DSC curves of Sample 6 from 70.0 °C to 110.0 °C with a rate of 5.0 °C/min. The red one is the heating curve and the blace one is the cooling curve.

Supplementary Note 6

Wide-angle X-ray diffraction of Sample 6 from 100.0 °C to −190.0 °C





Supplementary Figure 35 | WAXD profiles of Sample 6 from 100.0 °C to −190.0 °C. The red line is the blank curve and all curves are obained upon a cooling procedure.

Supplementary Note 7

The beneficial effect of TTPF to the BP temperature range in SLC-X

A commercial SLC-X (SLC Co. Ltd. Δn = 0.235, Δε =29.6 at 25 °C) was used as the host LC, and the concentration of chiral dopant BDH 1281 was fixed at 10.0 wt%. The dependence of BPs range on the concentration of TTPF concentration in the LCs mixture is shown in Fig. S10, the temperature range of BPs could be extended from 1.0 °C to over 15.5 °C.

Supplementary Figure 36 | Phase diagram of the SLC-X mixture. Dependence of BPs ranges on the concentration of TTPF with a fixed 10.0 wt% BDH1281 in the LCs mixture.

Supplementary References

1. Suzuki, A. Recent advances in the cross-coupling reactions of organoboron derivatives with organic electrophiles, 1995–1998. *J. Organomet. Chem.* **576**, 147-168 (1999).

2. Kotha, S., Lahiri, K. & Kashinath, D. Recent applications of the Suzuki–Miyaura cross-coupling reaction in organic synthesis. *Tetrahedron* **58**, 9633-9695 (2002).

3. Donaldson, T. *et al.* Symmetric and non-symmetric chiral liquid crystal dimers. *Liquid Crystals* **37**, 1097-1110 (2010).

4. Dierking, I. *Textures of Liquid Crystals* (Wiley, Weinheim, 2003).

5. Kikuchi, H., Izena, S., Higuchi, H., Okumura, Y. & Higashiguchi, K. A giant polymer lattice in a polymer-stabilized blue phase liquid crystal. *Soft Matter* **11**, 4572-4575 (2015).
