# Supplementary material for: Ultrastable liquid crystalline blue phase from molecular synergistic self-assembly
Source: Nat Commun. 2021 Mar 4;12:1440. doi: 10.1038/s41467-021-21564-y (PMC7933424; doi:10.1038/s41467-021-21564-y)
Supplement: Supplementary file 2 — Description of Additional Supplementary File [file 41467_2021_21564_MOESM2_ESM.docx]

**Description of Additional Supplementary File**

File Name: Supplementary Movie 1

Description: The video of optical textures of Sample 6 on cooling. Sample 6 was filled into a 20.0 μm-thick cell with no alignment treatment, and its textures were observed by POM. The video of optical textures of Sample 6 on cooling at first from 95.0 °C to 90.0 °C with a rate of 0.5 °C/min. and then from 90.0 °C to −193.5 °C at a rate of 2.0 °C/min was shown in Supplementary Movie 1.
